# The Effect of High Carbohydrate-to-fat Intake Ratios on Hypo-HDL-cholesterolemia Risk and HDL-cholesterol Levels over a 12-year Follow-up

**DOI:** 10.1038/s41598-020-57931-w

**Published:** 2020-01-22

**Authors:** Hye Ah Lee, Hyoin An

**Affiliations:** 1 Clinical Trial Center, Mokdong Hospital, Ewha Womans University, Seoul, Korea; 2 Department of Statistics, Ewha Womans University, Seoul, Korea

**Keywords:** Dyslipidaemias, Risk factors

## Abstract

Considering the strong correlation between carbohydrate and fat intake, we defined and assessed the association of the carbohydrate-to-fat ratio with the high-density lipoprotein cholesterol (HDL-c) level using 12-year follow-up data from the community-based cohort of the Korean Genome Epidemiology Study. We evaluated the long-term changes in HDL-c levels according to quartiles of carbohydrate-to-fat ratio using a mixed model. We also assessed the effect of the carbohydrate-to-fat ratio on the prevalence and incidence of hypo-HDL-cholesterolemia. Of 6,627 subjects, the prevalence of undiagnosed hypo-HDL-cholesterolemia at baseline was 35.3% (n = 2,339). Among the disease-free subjects, 56.8% developed hypo-HDL-cholesterolemia (incidence = 92/1,000 person-years). The prevalence and incidence of hypo-HDL-cholesterolemia were higher in females than in males. The highest carbohydrate-to-fat ratio quartile, which was characterized by high and low intake of carbohydrate and fat, was consistently associated with a lower HDL-c level during the 12-year follow up. Moreover, those in the highest quartile had a 1.14-fold greater risk of incident hypo-HDL-cholesterolemia than those in the lowest quartile, with a significant dose-response relationship. We found that high and low intake of carbohydrate and fat, respectively, was consistently associated with a low HDL-c level over a prolonged period. More research is needed to promote appropriate intake of macronutrients.

## Introduction

High-density lipoprotein (HDL) is known as good cholesterol because it helps to remove excess cholesterol from cells and atheroma^1^. Since the Framingham Heart Study reported a strong inverse association between the HDL-cholesterol (HDL-c) level and coronary heart disease^2^, many prospective studies have confirmed that HDL-c levels is a potential risk factor of cardiovascular disease (CVD)^3,4^, a leading cause of death.

In the 2010 Korea National Health and Nutrition Survey (KNHANES), one in two adults ≥20 years of age had dyslipidemia and hypo-HDL-cholesterolemia was the most common type (41.6%; 34.1% in males and 48.9% in females)^5^. These values are higher than those reported in Western countries^6,7^. In this regard, it has been suggested that a traditional dietary pattern, which is characterized by a high intake of carbohydrate, contributes to hypo-HDL-cholesterolemia in Koreans^8^.

Regarding the relationship between carbohydrate and lipid levels, several meta-analyses of randomized controlled clinical trials have shown that a low intake of carbohydrate improves the blood HDL-c level^9,10^. A Chinese population cohort study also reported that high intake of carbohydrate was associated with a decreased HDL-c level and increased triglyceride level during a 4.2-year follow up^11^. However, there is a seesaw effect between carbohydrate and fat intake: as the intake of one increases, that of the other decreases^12^. Indeed, carbohydrate intake influences risks to health in the direction opposite that of fat intake. This has hampered determination of the net effect of carbohydrate or fat on health risks. Moreover, few studies have assessed longitudinal changes in lipid levels according to intake of carbohydrate and fat.

Therefore, focusing on the high prevalence of hypo-HDL-cholesterolemia in Korea, we defined the carbohydrate-to-fat ratio and evaluated its association with changes in HDL-c levels using data from a Korean cohort study with a 12-year follow up. We also estimated the effect of the carbohydrate-to-fat ratio on the risk of hypo-HDL-cholesterolemia.

## Results

The baseline characteristics of the subjects are listed in Table 1. More than half of the subjects were females. The average BMI was 24.3 kg/m^2^ and 39.0% of the subjects were obese. The daily total energy intake was 1,950.1 kcal and 70.9% of total energy was derived from carbohydrate. With the exception of the age distribution, the baseline characteristics differed according to sex and half of the male subjects were current smokers at baseline.

**Table 1:** Characteristics of the subjects at baseline.

Characteristic	Total	Males	Females	*p*
	(n = 6,627)	(n = 3,193, 48.2%)	(n = 3,434, 51.8%)	
Age	51.15 ± 8.69	51.05 ± 8.61	51.25 ± 8.77	0.37
40–49 years	3495 (52.74)	1703 (53.34)	1792 (52.18)	0.27
50–59 years	1653 (24.94)	805 (25.21)	848 (24.69)	
60–69 years	1479 (22.32)	685 (21.45)	794 (23.12)	
Rural residence	3,200 (48.29)	1,459 (45.69)	1,741 (50.70)	<0.0001
Education level				
Less than high school	3,576 (54.23)	1,355 (42.60)	2,221 (65.07)	<0.0001
Graduated high school	2,123 (32.20)	1,154 (36.28)	969 (28.39)	
Some college or higher	895 (13.57)	672 (21.13)	223 (6.53)	
BMI (kg/m^2^)	24.31 ± 3.07	24.05 ± 2.92	24.56 ± 3.19	<0.0001
Normal (<23 kg/m^2^)	2,251 (33.97)	1,140 (35.74)	1,111 (32.38)	0.001
Overweight (23–24.9 kg/m^2^)	1,793 (27.06)	877 (27.49)	916 (26.70)	
Obese (≥25 kg/m^2^)	2,582 (38.97)	1,175 (36.77)	1,407 (40.92)	
Physical activity^1^ (MET-hours/week)	138.25 (79.63–249.38)	141.75 (78.75–259.00)	132.56 (81.38–229.25)	<0.0001
Q1 (<25th)	1,658 (25.02)	805 (25.21)	853 (24.84)	<0.001
Q2 (25–49th)	1,661 (25.06)	739 (23.14)	922 (26.85)	
Q3 (50–74th)	1,653 (24.94)	787 (24.65)	866 (25.22)	
Q4 (≥75th)	1,655 (24.97)	862 (27.00)	793 (23.09)	
Alcohol intake (g/day)	0 (0.00–8.68)	7.88 (0.00–24.63)	0 (0.00–0.29)	<0.0001
None	3348 (51.77)	886 (28.56)	2462 (73.16)	<0.0001
<15.0 g/day	1872 (28.95)	1039 (33.49)	833 (24.75)	
15.0–24.9 g/day	448 (6.93)	409 (13.19)	39 (1.16)	
≥25.0 g/day	799 (12.36)	768 (24.76)	31 (0.92)	
Current smoking (yes)	1,730 (26.33)	1,611 (50.58)	119 (3.52)	<0.0001
Macronutrients				
Energy (kcal/day)	1950.05 ± 617.54	2015.94 ± 592.97	1888.79 ± 633.51	<0.0001
CHO (g/day)	343.06 ± 105.98	348.80 ± 98.60	337.70 ± 112.20	<0.0001
% of total energy from CHO	70.93 ± 6.92	69.80 ± 6.71	71.98 ± 6.96	<0.0001
Fat (g/day)	32.63 ± 18.54	35.82 ± 18.80	29.69 ± 17.79	<0.0001
% of total energy from fat	14.56 ± 5.36	15.47 ± 5.18	13.71 ± 5.38	<0.0001
Protein (g/day)	66.20 ± 26.12	69.02 ± 25.78	63.57 ± 26.17	<0.0001
% of total energy from protein	13.45 ± 2.33	13.55 ± 2.27	13.35 ± 2.38	<0.001

CHO, Carbohydrate; BMI, body mass index; HDL, high-density lipoprotein; MET, metabolic equivalent of task

The median values of the carbohydrate-to-fat ratio according to quartile were 6.9, 9.5, 12.1, and 16.8, respectively. The distribution of baseline characteristics differed according to carbohydrate-to-fat ratio quartile. The subjects in the highest quartile were more likely to be female and older, have a lower education level, and live in a rural area compared with those in the lowest quartile. The average daily intake of carbohydrate and of fat of the highest quartile was 404.8 g (78.2% of total energy) and 22.1 g (9.0% of total energy), respectively (Table 2).

**Table 2:** Baseline characteristics of the subjects according to carbohydrate-to-fat ratio quartile.

	Carbohydrate-to-fat ratio quartile				*P*
	Q1 (<8.3)	Q2 (8.3–10.7)	Q3 (10.8–13.6)	Q4 (≥13.7)	
no. subjects	1,657	1,657	1,657	1,656	
Male	914 (55.16)	877 (52.93)	801 (48.34)	601 (36.29)	<0.0001
Age	48.34 ± 7.62	49.60 ± 8.09	51.38 ± 8.49	55.3 ± 8.90	<0.0001
40–49 years	1,121 (67.65)	1,001 (60.41)	833 (50.27)	540 (32.61)	<0.0001
50–59 years	338 (20.40)	386 (23.30)	457 (27.58)	472 (28.50)	
60–69 years	198 (11.95)	270 (16.29)	367 (22.15)	644 (38.89)	
Rural residence	580 (35.00)	558 (33.68)	775 (46.77)	1,287 (77.72)	<0.0001
Education level					
Less than high school	640 (38.76)	748 (45.33)	952 (57.73)	1236 (75.18)	<0.0001
Graduated high school	681 (41.25)	627 (38.00)	510 (30.93)	305 (18.55)	
Some college or higher	330 (19.99)	275 (16.67)	187 (11.34)	103 (6.27)	
BMI (kg/m^2^)	24.29 ± 3.04	24.33 ± 2.94	24.26 ± 3.00	24.38 ± 3.30	0.67
Normal (<23 kg/m^2^)	552 (33.39)	555 (33.49)	568 (34.30)	576 (34.80)	0.37
Overweight (23–24.9 kg/m^2^)	481 (29.10)	454 (27.40)	438 (26.45)	420 (25.38)	
Obese (≥25 kg/m^2^)	620 (37.51)	648 (39.11)	650 (39.25)	659 (39.82)	
Physical activity^1^ (MET-hours/week)	131.25 (78.75–199.50)	131.25 (78.75–201.25)	134.75 (81.38–244.13)	170.63 (84.00–287.00)	<0.0001
Q1 (<25th)	418 (25.23)	429 (25.89)	410 (24.74)	401 (24.21)	<0.0001
Q2 (25–49th)	471 (28.42)	468 (28.24)	431 (26.01)	291 (17.57)	
Q3 (50–74th)	466 (28.12)	440 (26.55)	425 (25.65)	322 (19.44)	
Q4 (≥75th)	302 (18.23)	320 (19.31)	391 (23.60)	642 (38.77)	
Alcohol intake (g/day)	1.65 (0–16.79)	0.55 (0–11.58)	0 (0–7.24)	0 (0–2.17)	<0.0001
None	656 (40.69)	777 (47.67)	884 (54.33)	1031 (64.52)	<0.0001
<15.0 g/day	537 (33.31)	501 (30.74)	457 (28.09)	377 (23.59)	
15.0–24.9 g/day	136 (8.44)	124 (7.61)	120 (7.38)	68 (4.26)	
≥25.0 g/day	283 (17.56)	228 (13.99)	166 (10.2)	122 (7.63)	
Current smoking (yes)	505 (30.72)	467 (28.27)	417 (25.30)	341 (20.97)	<0.0001
Macronutrients					
Energy (kcal/day)	2092.35 ± 701.33	1849.76 ± 487.33	1773.46 ± 438.92	2084.71 ± 724.25	<0.0001
CHO (g/day)	322.98 ± 104.60	319.41 ± 77.58	325.08 ± 70.71	404.82 ± 133.75	<0.0001
% of total energy from CHO	62.15 ± 4.91	69.55 ± 2.80	73.81 ± 2.78	78.20 ± 3.46	<0.0001
Fat (g/day)	50.70 ± 21.43	32.88 ± 11.83	24.92 ± 10.78	22.05 ± 12.73	<0.0001
% of total energy from fat	21.45 ± 3.63	15.57 ± 2.07	12.18 ± 2.22	9.02 ± 2.73	<0.0001
Protein (g/day)	82.41 ± 31.54	64.56 ± 20.15	56.86 ± 18.10	60.96 ± 24.94	<0.0001
% of total energy from protein	15.71 ± 2.19	13.86 ± 1.64	12.68 ± 1.55	11.54 ± 1.53	<0.0001

CHO, Carbohydrate; BMI, body mass index; MET, metabolic equivalent of task

The average HDL-c level in males was lower than that in females at baseline (48.2 mg/dL in males and 51.8 mg/dL in females), while the prevalence of hypo-HDL-cholesterolemia was higher in females than in males (68.5% in females *vs*. 31.6% in males, *p* < 0.0001). There was no difference in baseline HDL-c level according to the carbohydrate-to-fat ratio quartile, but prevalent hypo-HDL-cholesterolemia was positively associated with the carbohydrate-to-fat ratio. Even after adjusting for various covariates, the higher the quartile of the ratio, the higher the risk of hypo-HDL-cholesterolemia. This trend was observed in females, but not in males, and there was no interaction effect (*p* for interaction >0.05) (Table 3).

**Table 3:** Effect of carbohydrate-to-fat ratio quartile on the baseline HDL-c level and the prevalence of hypo-HDL-cholesterolemia.

	Total	Carbohydrate-to-fat ratio quartile				*P*
		Q1 (<8.3)	Q2 (8.3–10.7)	Q3 (10.8–13.6)	Q4 (≥13.7)	
Total (no. subjects)	6,627	1,657	1,657	1,657	1,656	
HDL Cholesterol (mg/dL)	50.04 ± 11.81	50.53 ± 11.81	50.08 ± 11.95	49.54 ± 11.60	50.03 ± 11.88	0.12
Prevalent hypo-HDL-cholesterolemia, n (%)	2339 (35.30)	510 (21.80)	554 (23.69)	610 (26.08)	665 (28.43)	<0.001
OR (95% CI)		1.00	1.03 (0.88–1.21)	1.13 (0.96–1.32)	1.16 (0.98–1.37)	<0.05^†^
Males (no. subjects)	3,193	914	877	801	601	
HDL Cholesterol (mg/dL)	48.17 ± 11.60	48.36 ± 11.32	47.99 ± 11.49	47.75 ± 11.52	48.73 ± 12.28	0.41
Prevalent hypo-HDL-cholesterolemia, n (%)	738 (31.55)	206 (27.91)	206 (27.91)	190 (25.75)	136 (18.43)	0.92
OR (95% CI)		1.00	1.03 (0.81–1.30)	1.10 (0.86–1.41)	1.07 (0.81–1.41)	0.51^†^
Females (no. subjects)	3,434	743	780	856	1,055	
HDL Cholesterol (mg/dL)	51.78 ± 11.75	53.21 ± 11.86	52.43 ± 12.03	51.21 ± 11.43	50.77 ± 11.59	<0.001
Prevalent hypo-HDL-cholesterolemia, n (%)	1601(68.45)	304 (18.99)	348 (21.74)	420 (26.23)	529 (33.04)	<0.001
OR (95% CI)		1.00	1.06 (0.86–1.31)	1.19 (0.96–1.47)	1.20 (0.96–1.48)	0.06^†^

HDL, high-density lipoprotein; OR, odds ratio; 95% CI, 95% confidence interval.

^†^*p* for trend.

Odds ratios with 95% confidence intervals were calculated after adjusting for sex, age, rural residence, education level, BMI (normal, overweight, and obese), physical activity quartile, alcohol intake, current smoking, and total energy intake. In the stratified analysis by sex, the variable sex was naturally excluded from the covariates.

**Figure 1:**
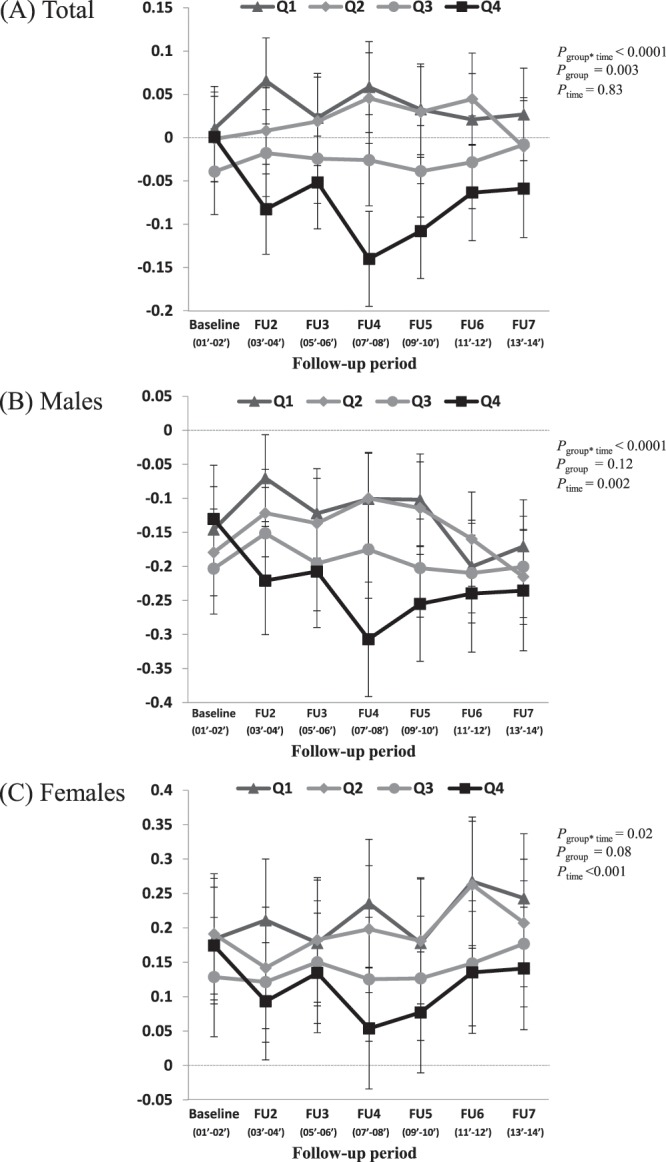
Change in HDL-c levels from baseline to the final follow-up according to carbohydrate-to-fat ratio quartile. High-density lipoprotein cholesterol (HDL-c) levels were transformed to standardized values based on the mean and standard deviation of HDL-c levels s of the subjects who participated in each follow-up survey. Values are least-squared means with 95% confidence intervals. The least-squared mean change in HDL-c levels was estimated for each carbohydrate-to-fat ratio quartile at each follow up using a mixed model assuming a random intercept with a compound symmetric structure. Estimates were obtained from a model that included the quartile of carbohydrate-to-fat ratio, follow-up time point, sex, age, rural residence, education level, physical activity, total energy intake at baseline, current smoking (at each follow-up), alcohol intake (at each follow up), body mass index (at each follow up), and the interaction between carbohydrate-to-fat ratio quartile and follow-up time point. In the stratified analysis by sex, the variable sex was naturally excluded from the covariates.

Longitudinal changes in HDL-c levels according to carbohydrate-to-fat ratio quartile are shown in Fig. 1. During the follow-up period, the average HDL-c level was high for the first quartile (relatively low intake of carbohydrate and high intake of fat), and low for the fourth quartile; this tendency was evident in both sexes. Additionally, the difference in HDL-c levels between the top and bottom quartiles (Q4 *vs*. Q1) in all subjects was significant after baseline (*p* < 0.05), even after controlling for covariates (Fig. 1). The longitudinal changes in HDL-c levels according to the carbohydrate-to-fat ratio quartile were similar to those according to the carbohydrate intake quartile (Supplemental Fig. 1).

Among the disease-free subjects (n = 4,288), 56.8% developed hypo-HDL-cholesterolemia (n = 2,435, incidence = 92 per 1,000 person-years). The incidence of hypo-HDL-cholesterolemia was higher in females than in males (128 per 1,000 person-years in females *vs*. 71 per 1,000 person-years in males). Compared to the first quartile (Q1) of the carbohydrate-to-fat ratio, the risk of hypo-HDL-cholesterolemia for Q2, Q3, and Q4 was 1.08 (95% CI 0.96–1.21), 1.21 (95% CI 1.08–1.35), and 1.47 (95% CI 1.32–1.65), respectively. After adjusting for covariates, the effect of carbohydrate-to-fat ratio quartile was weakened but the trend was maintained. The dose-response relationship remained even after controlling for fiber intake and fiber was not independently associated with incident hypo-HDL-cholesterolemia (Table 4). The carbohydrate-to-fat ratio quartile exerted the dominant effect on incident hypo-HDL-cholesterolemia in females, but there was no interaction effect (*p* for interaction = 0.53, Supplemental Table 1). In addition, the effects of the interactions of the carbohydrate-to-fat ratio quartile with other risk factors were non-significant (Supplemental Table 1). Use of the carbohydrate intake quartiles yielded similar results, while those of the fat intake quartiles were in the opposite direction (Supplemental Table 2).

**Table 4:** Effect of carbohydrate-to-fat ratio quartile on the incidence of hypo-HDL-cholesterolemia.

Parameter	Carbohydrate-to-fat ratio quartile				*p*_trend_
	Q1 (<8.3)	Q2 (8.3–10.7)	Q3 (10.8–13.6)	Q4 (≥13.7)	
Total (no. subjects)	1,147	1,103	1,047	991	
Cases/person-years	591/7656	590/6996	614/6381	640/5405	
Univariate	1.00	1.08 (0.96–1.21)	1.21 (1.08–1.35)	1.47 (1.32–1.65)	<0.0001
Model 1	1.00	1.04 (0.92–1.16)	1.10 (0.98, 1.24)	1.14 (1.01, 1.29)	0.02
Model 2	1.00	1.03 (0.91, 1.15)	1.09 (0.97, 1.22)	1.12 (0.99, 1.27)	0.04
Male (no. subjects)	708	671	611	465	
Cases/person-years	327/5050	306/4672	310/4123	248/2926	
Univariate	1.00	1.00 (0.85–1.17)	1.13 (0.96–1.32)	1.27 (1.08–1.50)	0.002
Model 1	1.00	0.96 (0.82–1.13)	1.05 (0.89–1.23)	1.07 (0.90–1.27)	0.34
Model 2	1.00	0.95 (0.81–1.12)	1.04 (0.88–1.22)	1.06 (0.88–1.26)	0.42
Female (no. subjects)	439	432	436	526	
Cases/person-years	264/2606	284/2324	304/2258	392/2479	
Univariate	1.00	1.19 (1.00–1.41)	1.28 (1.08–1.51)	1.49 (1.28–1.75)	<0.0001
Model 1	1.00	1.14 (0.96–1.35)	1.17 (0.98–1.39)	1.22 (1.03–1.45)	0.03
Model 2	1.00	1.13 (0.95–1.34)	1.16 (0.97–1.37)	1.20 (1.01–1.43)	<0.05

HDL, high-density lipoprotein.

Results are hazard ratios with 95% confidence intervals.

Hazard ratios of Model 1 were calculated with adjustment for sex, age, rural residence, education level, BMI (normal, overweight, and obese), physical activity quartile, alcohol intake, current smoking, and total energy intake.

Model 2: Model 1 + fiber intake.

In the stratified analysis by sex, the variable sex was naturally excluded from the covariates.

## Discussion

Using long-term follow-up data, we evaluated the effect of carbohydrate-to-fat ratio on the longitudinal changes in HDL-c levels and the risk of hypo-HDL-cholesterolemia. The highest carbohydrate-to-fat ratio quartile, which was characterized by high carbohydrate and low fat intake, was consistently associated with a lower HDL-c level during the 12-year follow up. Moreover, the carbohydrate-to-fat ratio was associated with prevalent and incident hypo-HDL-cholesterolemia; subjects in the top quartile had a 1.14-fold (95% CI 1.01–1.29) greater risk of incident hypo-HDL-cholesterolemia than those in the bottom quartile, with a significant dose-response relationship. The carbohydrate-to-fat ratio exerted a dominant effect in females, and there was no difference according to sex.

Most studies evaluated the effect of the intake of carbohydrate or fat individually. A cross-sectional South Korean study evaluated the association with metabolic syndrome of the carbohydrate and fat intake tertiles in combination; however, the subjects were not evenly distributed^13^. To consider the two highly correlated nutrients together, we calculated the carbohydrate-to-fat ratio and applied it. Irrespective of the total energy intake, if the intake of carbohydrate is 13.7-fold higher than that of fat, the risk of incident hypo-HDL-cholesterolemia increases by 14% (HR 1.14, 95% CI 1.01–1.29). Additionally, the results based on quartiles of carbohydrate intake were similar to those based on the carbohydrate-to-fat ratio; individuals in the highest carbohydrate intake quartile had a higher risk of hypo-HDL-cholesterol. Taken together, people who ate more than 363 g of carbohydrate (72.5% of 2,000 kcal), while eating less than 26.5 g of fat (11.9% of 2,000 kcal) per day were likely to be at high risk of developing hypo-HDL-cholesterolemia. Although its validity needs to be confirmed, assessment based on a ratio value overcomes the disadvantages of a previous study (i.e., the limitation in assessment associated with the unbalanced distribution of subjects when assessed as a group for combinations of intakes of two strongly correlated nutrients^13^), and was directly comparable to the intake of fat or carbohydrate.

Regarding the effect of carbohydrate intake on HDL-c levels, most studies have reported a significant association. Population-based cross-sectional South Korea studies reported that a high intake of carbohydrate is associated with an increased risk of hypo-HDL-cholesterolemia^14^ and metabolic syndrome^13^. Similar results have been reported from China^11^ and Japan^15^. In the cross-sectional PURE study, which involved 18 countries, the HDL-c, total cholesterol, and low-density lipoprotein cholesterol levels tended to decrease with increasing carbohydrate intake^16^. Similarly, a meta-analysis of 23 trial studies found that a low-carbohydrate diet (≤45% of energy from carbohydrates) improved the lipid level during a 6-month follow up (increase of 4.5 mg/dL in HDL-c levels and decrease of 30.4 mg/dL in the triglyceride level)^9^. In addition, a low-carbohydrate diet had a greater impact on lipid levels than did a low-fat diet (≤30% of energy from fat)^9^. In line with prior reports, our findings indicate an inverse association between carbohydrate intake and HDL-c levels. Additionally, the mixed-model analysis showed that the effect of diet on HDL-c levels was maintained over a long period. Although many studies have been conducted, our findings are meaningful in that they were obtained through long-term observation.

A high intake of carbohydrate has an unfavorable effect on health, but it is generally recommended to reduce the intake of fat or saturated fat rather than that of carbohydrate^12^. This is largely based on studies involving North American and European subjects, who have high intakes of fat and saturated fat^12,16^. However, due to the seesaw effect between carbohydrate and fat intake, a low-fat diet may exert an unfavorable effect on lipid levels. In addition, the benefit of replacing fat with other nutrients is controversial. A multinational cross-sectional study reported that replacement of saturated fat with carbohydrates exerted an adverse effect on lipid levels^16^. Two large cohort studies failed to show a link between replacement of saturated fat with carbohydrate and a decreased risk of CVD^17^. A recent study of the Atherosclerosis Risk in Communities (ARIC) cohort in the US reported that carbohydrate intake and mortality showed a U-shaped association, and the risk of mortality was lowest at a carbohydrate proportion of 50–55%^18^. Therefore, further research using data of the population of interest is needed.

In the 2015 Korean dietary guidelines, carbohydrate and fat are recommended to contribute 55–65% and 15–30% of total energy, respectively^19^. The contribution of carbohydrate to total energy has steadily declined over the past few decades^20^, but the average in Korea is higher than in the US (63.6% in Korea in 2016 *vs*. 48.6% in the US during 2011–2014)^20,21^. The main source of carbohydrate for Koreans is white rice, which is a refined grain and accounts for about 40% of the total carbohydrate intake^20^. Refined grain has a high glycemic index (GI) and glycemic load (GL), which lead to an elevated insulin response. In addition, foods with a high GI and GL are linked to metabolic processes such as lipolysis, lipogenesis, or substrate oxidation^22^, which cause endothelial damage and vascular dysfunction^23^. In Koreans, intake of refined grain may be related to a low HDL-c level. The subjects with a high carbohydrate-to-fat ratio were older and more likely to reside in a rural area, and to have a low education level. Sociodemographic factors also seem to have contributed to the association between diet and lipid level. There was a significant association between the carbohydrate-to-fat ratio and HDL-c level in females, but not in males, but the results of the mixed model consistently showed a low HDL-c level in the highest quartiles of the carbohydrate-to-fat ratio in both sexes. Thus, a high carbohydrate-to-fat ratio appears to have some effect on HDL-c levels, but the effects evaluated in terms of only the clinical definition (i.e. hypo-HDL-cholesterol) appeared to differ between males and females.

Several points should be considered when interpreting our results. First, the KoGES community-based cohort is not representative of the total population of South Korea. We did not take into account changes in the intake of macronutrients, which could have influenced the association. Dietary data were collected using a validated tool by dieticians, but measurement errors could dilute the association between the carbohydrate-to-fat ratio and hypo-HDL-cholesterolemia. There is also the possibility of bias due to loss to follow-up. Finally, although we analyzed several covariates, residual confounding factors, such as supplements, might have influenced the results.

This study had several strengths. The data were derived from a large-scale, long-term observational study conducted in Asia. Because of the possibility of dietary control for disease management, we excluded subjects with physician-diagnosed diseases at baseline. Repeated-measurement data were used to control for within-person variability. Changes in measurement settings are inevitable in a long-term observational study, so we used standardized values in the mixed-model analysis. It helped to satisfy the assumptions of the analysis and show a clear relationship.

In summary, we assessed the association of the carbohydrate-to-fat ratio with HDL-c levels. A high intake of carbohydrate and low intake of fat influenced HDL-c levels over a long period. The carbohydrate-to-fat ratio was also associated with the prevalence and incidence of hypo-HDL-cholesterolemia, with a marked dose-response relationship. To reduce the prevalence of hypo-HDL-cholesterolemia, further research to promote appropriate intake of macronutrients is needed.

## Methods

### Study subjects

This study was conducted using data from the community-based cohort of the Korean Genome Epidemiology Study (KoGES). The KoGES community-based cohort was established in 2001–2002 and an eighth follow-up survey was conducted in 2017–2018. Subjects were randomly selected from among 40- to 69-year-old residents of Ansung and Ansan, Gyeonggi Province, and were contacted via mail, telephone, or home visits (Ansung, response rate = 69.6%; Ansan, response rate = 45.7%). A total of 10,030 volunteers (Ansung, n = 5,018; Ansan, n = 5,012) were enrolled and completed the baseline survey. Ansung is located in a rural region and Ansan in an industrial region. The follow up was performed by trained technicians and interviewers at 2-year intervals, and involved the assessment of demographic factors, disease history, and health-related behaviors using questionnaires, anthropometric measurements, and biomarker assays. All participants provided informed consent^24^. The KoGES data are available with approval from the National Research Institute of Health^24^. The most recent available data are from the sixth follow-up survey conducted in 2013–2014 (follow-up rate, 62.8%). Information about this cohort can be found elsewhere^24,25^.

Subjects who met any of the following criteria were excluded: missing dietary survey data at baseline (n = 326); a daily caloric intake of <500 kcal or >5,000 kcal (n = 84); a history of cancer of any type, myocardial infarction, stroke, coronary artery disease, congestive heart failure, dyslipidemia, hypertension, or diabetes diagnosed by a physician in the baseline survey (n = 2,990); and missing data for the baseline HDL-c level (n = 3). Thus, the data of 6,627 subjects (3,193 males and 3,434 females) were analyzed in this study. Compared to those included in this study, the excluded subjects were slightly older and had a higher body mass index (BMI) and lower physical activity level, but their frequency of current smoking, total energy intake, and macronutrient intake did not differ (data not shown). The study protocol was approved by the Institutional Review Board of Ewha Womans University Hospital (IRB no. EUMC 2017–06–041). All methods were performed in accordance with the relevant guidelines and regulations.

### Macronutrient intake

Average food intake during the past year was collected using a dish-based semi-quantitative food-frequency questionnaire (FFQ) by dieticians during the baseline survey. The FFQ consists of 103 food items and has acceptable validity and reliability^26^. The daily intake of nutrients was calculated based on weight, which was derived from the frequency of consumption and the portion size of each food item. The KoGES provides data on the intake of 24 nutrients, including total energy. Using the data on the daily intake of carbohydrate and fat (both in grams), we estimated the energy-adjusted intake of carbohydrate and fat using the residuals method and calculated the carbohydrate-to-fat ratio. The carbohydrate-to-fat ratio was divided into quartiles for use in subsequent analyses.

### Outcomes

The main outcome variable was HDL-c levels in blood during the follow up. Blood samples were collected after fasting overnight. HDL-c levels was measured using an autoanalyzer (ADVIA 1650 or 1680, Siemens, Tarrytown, NY, USA). To reduce variability due to measurement settings, HDL-c levels were transformed to standardized values using the mean and standard deviation of HDL-c levels from subjects who participated in each follow-up survey.

Hypo-HDL-cholesterolemia was defined as an HDL level of <40 mg/dL for males and of <50 mg/dL for females. Follow-up began upon entry into the study and ended on the date of detection of an abnormal value of a biochemical marker or of the final follow-up, whichever was sooner.

### Covariate factors

HDL-c levels tends to be lower in individuals with obesity, inflammation, and those who smoke, and tends to be higher in persons who exercise and consume alcohol^27^. Among the available data, we analyzed BMI, current smoking, alcohol intake, physical activity, and demographic factors. Education level was classified into did not graduate high school, graduated high school, or at least some college. BMI was categorized as normal (<23.0 kg/m^2^), overweight (23–24.9 kg/m^2^), or obese (≥25 kg/m^2^). The alcohol-intake categories were based on previous studies (no alcohol, <15 g/day, 15–24.9 g/day, and ≥25 g/day)^28,29^. Physical activity over the past year was assessed using the International Physical Activity Questionnaire^30^ and was measured in terms of the quartile of Metabolic Equivalent of Task (MET)-hours per week, which takes into account both the intensity and duration of exercise. We also considered the energy-adjusted intake of fiber.

### Statistical analysis

All statistical analyses were conducted using SAS ver. 9.4 (SAS Institute, Cary, NC, USA). Normally and non-normally distributed data are expressed as means with standard deviations and as medians with interquartile ranges, respectively; categorical data are presented as numbers of subjects with percentages. Differences in baseline characteristics by carbohydrate-to-fat ratio quartile were evaluated by analysis of variance and the Kruskal–Wallis test for numerical data and the chi-squared test for categorical data. We also evaluated the association between the carbohydrate-to-fat ratio quartile and prevalent hypo-HDL-cholesterolemia at baseline.

Longitudinal changes in HDL-c levels according to carbohydrate-to-fat ratio quartile were evaluated by mixed-model analysis assuming a random intercept model. The model included group (*i*.*e*., carbohydrate-to-fat ratio quartiles), follow-up time point, and the interaction between group and follow-up time point as fixed effects. Based on the low Akaike information criterion (AIC) values, a covariance structure was determined, and a compound symmetric covariance structure was used. Additionally, baseline characteristics (sex, age, region [rural/industrial], education level, and physical activity quartile) and repeated measurements data (BMI status, current smoking, and alcohol intake) were included as covariates. Results are presented as least-squared means with 95% confidence intervals (CIs).

For the analysis of incident hypo-HDL-cholesterolemia, we excluded a further 2,339 subjects with prevalent hypo-HDL-cholesterolemia at baseline. We calculated the incidence of hypo-HDL-cholesterolemia in units of 1,000 person-years. To assess the effect of the carbohydrate-to-fat ratio on the development of hypo-HDL-cholesterolemia, we estimated the HR with 95% CI using the Cox proportional hazards model. The assumption of the Cox proportional hazards model was evaluated using the Schoenfeld residuals method and was satisfied. The effect of the interactions between carbohydrate-to-fat ratio and the covariates at baseline was also evaluated, as was that of carbohydrate and fat intake on incident hypo-HDL-cholesterolemia. Statistical significance was determined at *p* < 0.05 in a two-tailed test.

## Supplementary information

Supplementary Information.
