# Supplementary material for: The Effect of High Carbohydrate-to-fat Intake Ratios on Hypo-HDL-cholesterolemia Risk and HDL-cholesterol Levels over a 12-year Follow-up
Source: Sci Rep. 2020 Jan 22;10:913. doi: 10.1038/s41598-020-57931-w (PMC6976611; doi:10.1038/s41598-020-57931-w)
Supplement: Supplementary file 1 — Supplementary Information. [file 41598_2020_57931_MOESM1_ESM.pdf]

The Effect of High Carbohydrate-to-fat Intake Ratios on Hypo-HDL-cholesterolemia Risk and HDL-cholesterol Levels over a 12-year Follow-up

Hye Ah Lee[1*], Hyoin An[2]

[1]Clinical Trial Center, Mokdong Hospital, Ewha Womans University, Seoul, Korea,
[2] Department of Statistics, Ewha Womans University, Seoul, Korea

Supplemental Table 1. Interaction effect of the carbohydrate-to-fat ratio quartile and baseline characteristics on incident hypo-HDL-cholesterolemia

	HR (95% CI)	*p* for Interaction with the carbohydrate-to-fat ratio quartile
Sex		
Male	ref	0.53
Female	1.52 (1.37-1.69)	
Age group		
40–49 years	ref	0.53
50–59 years	1.03 (0.93-1.14)	
60–69 years	1.09 (0.97-1.22)	
Rural region		
No	ref	0.72
Yes	1.31 (1.19-1.46)	
Education level		
Less than high school	ref	0.24
Graduated high school	0.97 (0.88-1.07)	
College or higher	0.86 (0.75-0.99)	
BMI status		
Normal (< 23 kg/m^2)	ref	0.25
Overweight (23–24.9 kg/m^2)	1.31 (1.19-1.45)	
Obese (≥ 25 kg/m^2)	1.37 (1.24-1.51)	
Physical activity quartile (MET-hours/week)		
Q1 (< 25th)	ref	0.41
Q2 (25–49th)	0.97 (0.86-1.08)	
Q3 (50–74th)	0.90 (0.80-1.01)	
Q4 (≥ 75th)	0.86 (0.76-0.97)	
Alcohol intake (g/day)		
None	ref	0.36
< 15.0 g/day	0.79 (0.72-0.87)	
15.0–24.9 g/day	0.66 (0.55-0.79)	
≥ 25.0 g/day	0.69 (0.60-0.79)	
Smoking status		
Non-smoking and ever-smoking	ref	0.34
Current smoking	1.18 (1.06-1.32)	

HR, hazard ratios; CI, confidence interval; HDL, high-density lipoprotein; BMI, body mass index; MET, metabolic equivalent of task.

Hazard ratios calculated with adjustment for sex, age, rural residence, education level, BMI (normal, overweight, and obese), physical activity quartile, alcohol intake, current smoking, and total energy intake.

Supplemental Table 2. Effect of carbohydrate or fat intake quartile on the development of hypo-HDL-cholesterolemia

	Intake quartile				P_{trend}
	Q1	Q2	Q3	Q4	
**Carbohydrate intake (g/d)**	<325g	325-345g	346-362g	≥363g	
Total (no. subjects)	1146	1110	1034	998	
Cases/person-years	585/7615	586/7195	612/6199	652/5430	
Model	1.00	1.02 (0.91-1.14)	1.12 (1.00-1.26)	1.18 (1.05-1.34)	0.003
Male (no. subjects)	700	672	599	484	
Cases/person-years	319/4972	293/4832	314/3960	265/3007	
Model	1.00	0.92 (0.78-1.08)	1.10 (0.93-1.29)	1.12 (0.94-1.33)	0.08
Female (no. subjects)	446	438	435	514	
Cases/person-years	266/2643	293/2363	298/2239	387/2422	
Model	1.00	1.15 (0.97-1.36)	1.15 (0.97-1.30)	1.25 (1.05-1.49)	0.02
**Fat intake (g/d)**	<8g	8-10g	11-13g	≥14g	
Total (no. subjects)	986	1050	1107	1145	
Cases/person-years	638/5376	612/6450	595/6935	590/7677	
Model	1.00	0.95 (0.84-1.06)	0.92 (0.82-1.04)	0.87 (0.77-0.99)	0.03
Male (no. subjects)	459	611	668	717	
Cases/person-years	243/2901	310/4145	308/4588	330/5138	
Model	1.00	0.99 (0.83-1.18)	0.94 (0.79-1.13)	0.94 (0.79-1.13)	0.43
Female (no. subjects)	527	439	439	428	
Cases/person-years	395/2475	302/2305	287/2347	260/2539	
Model	1.00	0.92 (0.78-1.08)	0.93 (0.78-1.09)	0.81 (0.68-0.96)	0.03

HDL, high-density lipoprotein

Results are hazard ratios with 95% confidence intervals.

Hazard ratios calculated with adjustment for sex, age, rural residence, education level, BMI (normal, overweight, and obese), physical activity quartile, alcohol intake, current smoking, and total energy intake.

In the stratified analysis by sex, the variable sex was naturally excluded from the covariates.

Supplemental Figure 1. Change in HDL-c levels from baseline to the final follow-up according to carbohydrate intake quartile

(A) Total

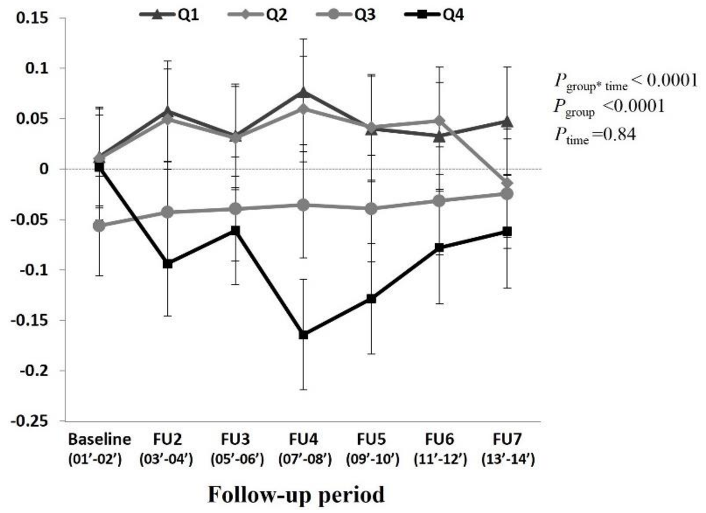

(B) Males

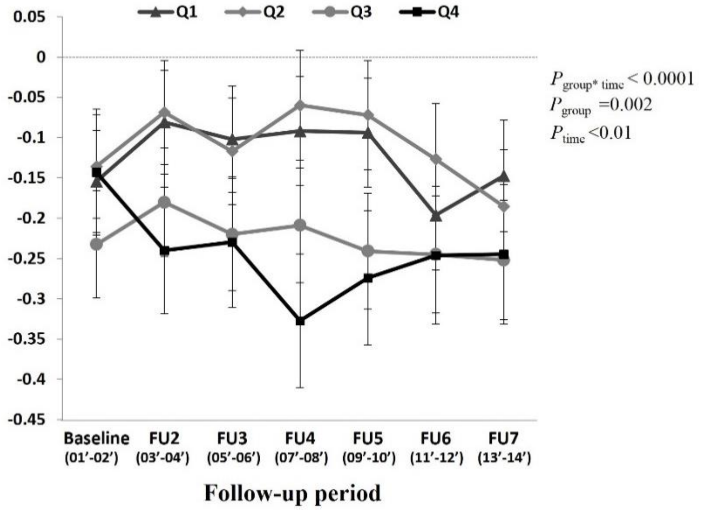

(C) Females

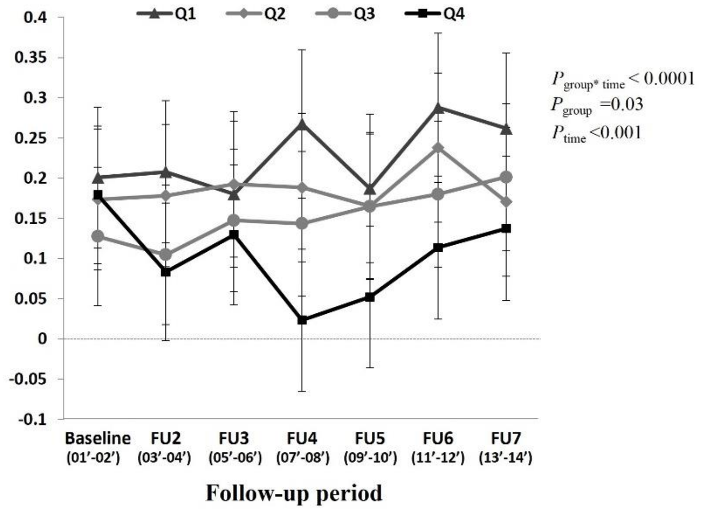

High-density lipoprotein cholesterol (HDL-c) levels were transformed to standardized values based on the mean and standard deviation of HDL-c levels s of the subjects who participated in each follow-up survey. Values are least-squared means with 95% confidence intervals. The least-squared mean change in HDL-c levels was estimated for each carbohydrate quartile at each follow up using a mixed model assuming a random intercept with a compound symmetric structure. Estimates were obtained from a model that included the quartile of carbohydrate, follow-up time point, sex, age, rural residence, education level, physical activity, total energy intake at baseline, current smoking (at each follow-up), alcohol intake (at each follow up), body mass index (at each follow up), and the interaction between carbohydrate quartile and follow-up time point. In the stratified analysis by sex, the variable sex was naturally excluded from the covariates.
